# Radioactive Molecules 2021–2022

**DOI:** 10.3390/molecules29010265

**Published:** 2024-01-04

**Authors:** Svend Borup Jensen

**Affiliations:** 1 Department of Nuclear Medicine, Aalborg University Hospital, 9000 Aalborg, Denmark; svbj@rn.dk; 2 Department of Chemistry and Biochemistry, Aalborg University, 9220 Aalborg, Denmark

In 2020 I was invited to write an editorial review on radioactive molecules published in *Molecules* in 2019 and 2020 [1]. The aim of the review was to identify all the papers published in the journal that applied radioactive isotopes. The present review has the same aim, but for the years 2021 and 2022. To make the data comparable, the articles are examined and categorized in the same manner as in the first review.

## 1. The Search Criteria

As in the previous editorial review, a search in *Molecules* was performed using the keyword “radio*”. In the 2021 to 2022 period, the search resulted in 276 articles, compared to 181 for 2019–2020, an increase of 52%.

A closer look at the identified articles revealed that 109 (60%) of those published in 2019–2020 were in fact dealing with radioactive isotopes, with this figure being 172 (62%) for 2021–2022. Again, approximately 40% of the articles from the search were not considered relevant to the review. I conducted an investigation into which search terms caused the articles not relevant to this review to appear in the search results and found that many of these articles deal with radioactive therapy and protection against radioactivity. The words found to occur more than 10 times in the 104 “irrelevant” articles were: radiotherapy, radioresistance/radio resistance, radiosensitivity, radio sensitization/radiosensitization, radiosensitizer(s), radio frequency/radiofrequency, and radiolysis.

Taking the above identified words into account, I attempted to apply othersearch terms to optimize the search, see Table 1. However, as can be seen from Table 1, the application of “radioa*”, “radioactive”, “isotope”, and “isotop*”, resulted in the search failing to find all of the relevant articles, so this search strategy was discarded.

## 2. Main Categories

### 2.1. Non-Medical Applications

Only 23 of the 172 selected articles pertained to non-medical subjects, typically dealing with pollution and nuclear waste. 

As an example of the subjects covered, two interesting papers dealing with the synthesis of compounds able to capture iodine [L1, L2]. In both papers, the aim was to develop compounds capable of removing radioactive iodine from nuclear power plant waste. The capture of iodine is, in fact, a hot research topic [2,3,4,5]. Compounds capable of removing radioactive iodine may also be medically relevant, as hospitals use iodine I-131 (with a half-life of 8 days) for treatment of some thyroid disorders, resulting in the patients urine becoming radioactive, resulting in it being categorized as radioactive waste. Hospitals handle this problem by using storage tanks for toilet waste to postpone its release into the environment and/or by mixing the toilet waste with other wastewater to dilute the radioactivity below the threshold level. Methods designed to extract I-131 from urine may indeed prove be more economically advantageous compared to storing toilet wastewater, especially if the iodine capture is reversible such that the extraction material can be re-used.

The other articles focusing on non-medical use of radioactive isotopes constitute a very diverse group. They cover a range of topics from the examination of naturally occurring isotopes by applying in vivo isotope degradation [L3], to thermo-degradation experiments [L4], and ^14^C-dating of a Roman Egyptian mummy portrait [L5].

### 2.2. Medical Applications

The number of articles covering medical uses of radioactive molecules was 149. Excluding my own editorial review [1] from 2021 leaves a total of 148 articles dealing, in one way or the other, with the medical uses of radioactive isotopes, to be further examined.

As in the previous review, the articles were divided into subgroups: diagnostics, therapy, synthesis, and nuclide preparation, and it was noted whether the article was a review (see Table 2). An article may appear in multiple subgroups.

Table 2 shows that the number of articles dealing with medical uses of radioactive isotopes has increased from 103 to 148 (an increase of 36%). In general, the use of radioactive molecules in clinical settings at hospitals has been increasing for many years, which can to some extent explain the rising research articles on this topic.

The percentage of reviews has increased from 20% to 28%. It is worth noting that all of the percentages have increased from 2019–2020 to 2021–2022. Part of the explanation for this is that the number of reviews has increased, and review articles often touch upon several topics such as diagnostics, therapy, synthesis, and nuclide preparation. However, the tendency of a greater number of articles covering topics from more than one of the subgroups cannot solely be put down to the increase in reviews. This increase supports a general sense I have when reading through the articles that, although an article focuses on, for example, synthesis, it also often contains information about how the radioactive substances are used in diagnostics and/or therapy. In other words, articles are increasingly covering more subjects (diagnostic, therapy, synthesis, and/or nuclide preparation) than was the case merely two years ago.

## 3. Use of Isotopes

The 148 articles on medical uses for radioactive isotopes, and the 23 on non-medical uses, deal with or mention many different isotopes, as can be seen in Table 3, Table 4, Table 5, Table 6 and Table 7.

The placement of the isotopes in Table 3, Table 4, Table 5, Table 6 and Table 7 depends on how the isotope mainly decays—i.e., whether the main form of its decay is via positron [6,7,8,9], gamma [10], beta [11], Auger [12], or alpha emissions [13]. The decay of an isotope will often take place in several ways; however, this review will focus on the main form of decay. The categorization of the isotopes and their location in Table 3, Table 4, Table 5, Table 6 and Table 7 follows the description of the isotopes primary decay in the reviewed articles, which moreover in broad terms are identical to what is found in the literature published elsewhere.

In Table 3 and Table 4, the articles are classified into three groups: diagnostic/use, synthesis/chelation, and isotope preparation. In Table 5, Table 6 and Table 7, the diagnostic column has been replaced by therapy/detection. Each article is placed in at least one of these groups but may appear in more than one group. The column entitled “Total Number of Articles” is the sum of the other columns but with duplicates subtracted.

The column “Selected Articles” lists articles that support and illustrate what I aim to express, furthermore it provide the reader with the opportunity to seek additional knowledge from the original literature.

As was the case with the 2021 review [1], ^18^F is the isotope mentioned in most articles. There has subsequently been an increase in papers mentioning ^18^F, but the increase has been much bigger for several of other isotopes. The second most used PET isotope in 2021/2022 was ^68^Ga, yet there were more than 2.5 as many articles on ^18^F than ^68^Ga in 2019/2020. At present, the numbers of articles covering these isotope are more closely aligned, there being only 1.16 more articles on ^18^F than ^68^Ga. The same trend is seen for a number of the other isotopes such as, for example, ^11^C, ^64^Cu, and ^89^Zr. The numbers of articles on ^11^C has risen from 6 to 26, on ^64^Cu the number is up from 2 to 33, and the number of articles on ^89^Zr has risen from 5 to 20. Furthermore, a significant number of new PET isotopes were discussed in the 2021–2022 issues of *Molecules*, which was not the case in 2019–2020; examples include ^13^N, ^22^Na, ^52^Mn, ^61^Cu, ^66^Ga, ^82^Rb, and ^86^Y.

There has been a considerable rise in the number of articles that mention gamma emitters. ^99m^Tc and ^125^I are mentioned approximately three times as often in 2021–2022 compared to 2019–2020. For the other isotopes, the increase is, in many cases, much higher. This supports my point that articles generally have an increased focus on explaining how their findings contribute to the general state of knowledge. In order to illustrate what their contribution means in a broader context, the authors must, to a higher degree, describe what other researchers have achieved. For that reason, a greater number of tracers and more isotopes are mentioned.

Additionally, there is an awareness of that developing and identifying the best possible tracer often means that a nuclide replacement is performed to unroll the full potential of a radioactive drug, this of course also contributes to more isotopes being mentioned in a given article.

As already mentioned, an isotope can often decay by more than one route; for example, ^177^Lu emits both beta and gamma radiation during its decay and can, therefore, be used for therapy as well as diagnostics. I have placed the isotopes in Table 3, Table 4, Table 5, Table 6 and Table 7 depending on how they are normally referred to. As ^177^Lu is normally referred to as a beta emitter, it is placed in Table 5.

A very interesting article by Stokke et al. [L18], mentions almost 100 different isotopes. For some of these, it was not clear in which of Table 3, Table 4, Table 5, Table 6 and Table 7 they should be placed. I have taken the liberty of not including those isotopes, as they are only mentioned in reference [L18] and they decay in several different ways—for example, as gamma and beta emitters, as well as by electron capture.

Theranostic is becoming more and more widespread in nuclear medicine. Theranostic is a two-stage process to diagnosing and treating cancers using radiotracers. The notion underpinning theranostics is that two almost identical radiopharmaceutical can first be used to diagnose/identify a cancer and then subsequently to treat the cancer. The difference between the diagnostic use and the therapeutic use is the replacement of the radioactive isotope: a positron or gamma emitter is used for diagnostics and, for treatment, a beta emitter, Auger electron emitter, or alpha emitter. It makes sense to know exactly where a beta, alpha, or Auger electron emitter goes before it is injected into a human subject.

## 4. Development of New Radiopharmaceuticals for Clinical Use

The development of new radiopharmaceuticals is performed in a similar manner to non-radioactive medicine, with synthesis followed by ex vivo examination, then in vivo animal examination, and finally by human clinical trials. Synthesis was discussed in 96 articles (65%, see Table 2). Following synthesis there is often some initial ex vivo testing of the radiopharmaceuticals. This was referred to in 58 of the 148 articles [L6, L8, L9, L10, L12, L16, L24, L25, L30, L31] on isotopes for medical use. Ex vivo tests are often simple stability tests of the compound or analysis to find log P (the partition coefficient: measuring how an analyte will partition between an aqueous and organic phase).

The next step is to perform in vivo tests with the radiopharmaceuticals in animals. Such tests are performed to examine the distribution of the radiopharmaceuticals, there in vivo stability, and in many cases, to examine their uptake in, for example, tumour cells. The preferred animals are mice: 51 articles covered the use of mice [L7–L13, L15, L16, L20, L24, L25, L28, L31, L32], 16 the use rats [L7, L13, L17, L20], 6 pigs [L7, L29], 3 baboons/monkeys, 2 rabbits, 1 dogs, and 1 sheep.

There were nine preliminary human studies and five clinical studies focusing on tracers. This is higher than in 2019–2020. The increase is probably due to more and more radiopharmaceuticals being approved for clinical trials. The amount of work a radiochemist must invest in a radiopharmaceutical being approved for clinical trials is significant, and that is probably also part of the reason why we see an increased number of publications on preliminary human studies focusing on the radiopharmaceuticals.

## 5. Diagnosing or Treating a Specific Disease

Of the 148 articles on isotopes for medical uses, 78 concerned cancers (Table 8), 24 brain disorders (Table 9), 15 inflammation and infection (Table 10), 15 other diseases, or which do not discuss a specific disease. The “other diseases” category includes, for example, liver diseases, diabetes mellitus, sclerosis, hyperthyroidism, myocardial injury, invasive fungal, renal protection, traumatic brain injury, spinal cord injury, and pulmonary disease. Also placed in this category are the articles which focus on imaging for, for example, lung perfusions, bone formation and bone imaging [L13], and sentinel lymph node detection. There was also one article on analgesia, one on liposomes, and one on the influence of caffeine on glucose uptake.

The column “Selected Articles” in Table 8, Table 9 and Table 10 lists articles that support and illustrate what I aim to express, furthermore they provide the reader with an opportunity to seek additional knowledge from the original literature.

Cancers is by far the largest category. In 2021–2022, there were 78 articles mentioning cancers, compared to 39 in 2019–2020; i.e., an increase of 61%. The most common cancer type in 2019–2020 was prostate with eight articles, which increased to 14 articles in 2021–2022. Although the number of articles dealing with prostate cancer increased by 75%, it was overtaken by other types of cancer when it comes to the number of articles published on a specific cancer type: for example, the number of articles on breast cancer increased from two articles in 2019–2020 to 20 articles in 2021–2022. Other significant increases were for bone cancer, which increased from 2 to 15, lung cancer which was up from 1 to 12, and liver cancer rising from 0 to 11. The reason why articles on prostate cancer did not increase as much as, for example, breast, liver, and lung cancer, is that many articles dealing with the PSMA analogue had already been published [14,15]. It is only natural that the research focus turned to other types of cancers where good radiopharmaceuticals are not yet as available as is the case for prostate cancer.

The increase in articles concerning brain disorders/brain function was by 33%, from 18 articles in 2019–2020 to 24 in 2021–2022. In 2019–2020, Alzheimer’s disease was the most mentioned subject with six articles, this increased to eight articles in 2021–2022. However, in 2021–2022, Alzheimer’s disease was overtaken by studies on Parkinson’s disease, which went up from 3 to 9 articles.

The increase of articles concerning inflammation/infection was by 114%, from 7 articles in 2019–2020 to 15 in 2021–2022. Bone infection/osteomyelitis increased from two articles in 2019–2020 to seven in 2021–2022, and neuroinflammation increased from one article in 2019–2020 to four in 2021–2022.

## 6. Overall Summary

In summary, the number of articles which apply radioactive isotopes in *Molecules* has increased by 58% over the last 2 years, from 109 articles in 2019–2020 to 176 articles in 2021–2022. Although a lot of interesting articles are being published in the area of non-medical research, articles with radioactive molecules are still mainly published in medicine for diagnosing or treating diseases. The number of different isotopes applied (see Table 3, Table 4, Table 5, Table 6 and Table 7) and the different diseases covered (see Table 8, Table 9 and Table 10) have increased considerably over the last two years.

## Figures and Tables

**Table 1:** Search terms applied.

	2019	2020	2021	2022
Radioa*	12	16	12	23
Radioactive	11	15	12	26
Isotope	46	42	51	56
Isotop*	46	42	50	54
Radio*	68	117	126	150
Relevant papers	109		78	94
Not relevant	76		48	56

**Table 2:** Division of the articles dealing with the medical use of isotopes into subgroups.

	Diagnostic	Therapy	Synthesis	Nuclide Preparation	Review	Articles Concerning Medical Use of Radioactive Isotopes
2019–2020	73 articles (71%)	24 articles (23%)	41 articles (40%)	9 articles (9%)	21 articles (20%)	103 articles (100%)
2021–2022	129 articles (87%)	57 articles (39%)	96 articles (65%)	21 articles (14%)	42 articles (28%)	148 articles (100%)

**Table 3:** Positron emitters.

	Total Number of Articles	Diagnostic	Synthesis/ Chelation	Isotope Preparation	Selected Articles
^11^C	26	26	9	-	[L6–L12]
^13^N	7	7	-	-	[L8, L9, L11, L13]
^15^O	7	7	-	-	[L7–L9, L13]
^18^F	59	57	25	-	[L6–L20]
^22^Na	2	2	-	-	-
^43^Sc	1	1	-	-	-
^44^Sc	10	8	5	3	[L20, L21]
^52^Mn	5	3	3	3	[L8, L20]
^55^Co	2	2	-	1	-
^61^Cu	2	2	-	1	[L13, L22]
^64^Cu	33	32	7	4	[L8, L9, L11, L13, L15, L18, L20, L22]
^66^Ga ^¤^		3	1	1	[L7]
^68^Ga	51	48	22	4	[L7–L9, L11, L13, L18–L20, L23–L25]
^82^Rb	3	3	-	-	[L9, L13]
^86^Y/^86g^Y	4	3	2	1	[L20]
^89^Zr	19	17	8	3	[L8, L13, L15, L20]
^124^I	13	11	6	1	[L8, L10, L13, L19, L26]
Positron emitter isotopes mentioned only in one article: ^43^Sc, ^152^Tb, ^44^Ti, ^76^Br, and ^120^I					

(^¤^ both a positron and a gamma emitter).

**Table 4:** Gamma emitters.

	Total Number of Articles	Diagnostic	Synthesis/ Chelation	Isotope Preparation	Selected Articles
^57^Co	2	2	-	1	[L20]
^62^Zn	2	2	-	-	[L18, L20]
^66^Ga	3	3	1	1	[L7]
^67^Ga ^¤^	10	10	2	1	[L7, L11, L13, L18, L22]
^99m^Tc	47	43	20	4	[L6, L8, L9, L13, L18, L20, L25, L27-L31]
^105^Rh	2	2	-	-	[L18]
^111^In	24	23	3	2	[L7, L9, L11, L13, L15, L18, L20, L24]
^123^I	16	14	4	-	[L8, L10, L11, L13, L18, L19, L26]
^125^I	18	17	6		[L8, L10, L13, L18, L26, L32, L33]
^133^Xe	2	2	-	-	[L13]
^201^Tl	2	2	-	-	[L13]
Isotopes mentioned only in one article: ^51^Cr [L13], ^51^Mn, and ^155^Tb. Reference [L19] is the only article to mention: ^76^As, ^77^Br, ^82^Br, ^86^Rb, ^90^Nb					

(^¤^ both a positron and gamma emitter).

**Table 5:** Beta emitters.

	Total Number of Articles	Therapy/ Detection	Synthesis/ Chelation	Isotope Preparation	Selected Articles
^3^H	15	14	5	-	[L9]
^14^C	1	1	-	-	-
^32^P	5	4	1	2	[L18, L34]
^35^S	3	3	1	1	-
^47^Sc	8	7	1	-	[L18, L20, L22]
^67^Cu *	9	8	2	1	[L18, L20, L22]
^89^Sr	3	3	-	-	[L18]
^90^Y	18	17	4	1	[L8, L13, L18, L25, L29, L34]
^117m^Sn	2	2	-	-	[L13, L18]
^131^I *	25	18	11	-	[L8, L13, L18, L19, L26, L29, L32, L34]
^153^Sm	9	9	-	1	[L13, L25, L34]
^161^Tb	3	3	-	-	[L18]
^166^Ho	4	4	1	-	[L13, L18, L29, L34]
^177^Lu	42	39	7	2	[L8, L13, L15, L18–L20, L22–L25, L34]
^186^Re	7	7	1	2	[L13, L18, L22, L34]
^188^Re	12	12	1	2	[L13, L18, L25, L29, L34]
^198^Au	3	3	-	1	[L18, L34]
Isotopes mentioned only in one article: ^14^C, ^33^P, ^90^Sr, ^129^I, ^210^Pb, ^169^Er [L34]					
Reference [L19] is the only article to mention: ^24^Na, ^33^P, ^54^Mn, ^76^As, ^77^As, ^77^Br, ^80^Br, ^91^Y, ^109^Pd, ^111^Ag, ^121^Sn, ^135^La, ^142^Pr, ^143^Pr, ^165^Dy, ^185^W, ^212^Pb					

* ^67^Cu and ^131^I are both beta and gamma emitters. One article mentions that ^67^Cu is also a gamma emitter and 10 articles mention that ^131^I is also a gamma emitter.

**Table 6:** Auger electron emitters.

	Total Number of Articles	Therapy	Synthesis/ Chelation	Isotope Preparation	Selected Articles
^58^Co/^58m^Co	2	2	-	1	[L18]
^165^Er	2	2	1	1	[L18]
^193m^Pt	2	2	1	-	[L18]
^195m^Pt	2	2	1	-	[L18]
Isotopes mentioned in only one article: ^114m^In, ^134^Ce [L18]					

**Table 7:** Alpha emitters.

	Number of Articles	Therapy	Synthesis/ Chelation	Isotopes Preparation	Selected Articles
^149^Tb	2	2	-	-	[L18]
^211^At	6	6	-	-	[L18, L34]
^212^Bi	2	2	-	-	[L18]
^213^Bi	5	4	1	-	[L18, L34]
^223^Ra	7	7	-	-	[L13, L18, L34]
^225^Ac	14	13	2	1	[L13, L15, L18, L25, L31, L34]
^227^Th	2	2	1	1	
Reference [L18] is the only article to mention: ^224^Ra, ^255^Fm					

**Table 8:** Cancers.

	Number of Articles	Selected Articles
All articles concerning cancer	78	[L8, L9, L12, L13, L15, L16, L18, L19, L24, L31, L32, L34]
Prostate cancer	14	[L8, L9, L12, L13, L31, L34]
Lymphoma (including B-cell)	2	[L8]
Neuroendocrine	13	[L8, L9, L13, L24]
Breast cancer	20	[L8, L9, L13, L31]
Gastric cancer	3	[L8]
Leukaemia	7	[L8, L12, L13, L34]
Renal cell carcinoma	4	[L8, L9]
Solid tumour/multiple myeloma	7	[L9, L12, L13]
Non-Hodgkin’s lymphoma	7	[L8, L9, L18, L34]
Pancreatic cancer	9	[L8, L9, L15, L24]
Ovarian cancer	12	[L9, L18, L24, L31, L34]
Bone-metastases, -pain, and -cancer	15	[L8, L9, L13, L18, L34]
Thyroid cancer	11	[L8, L9, L13, L19, L24, L34]
Non-small-cell lung cancer/lung cancer	12	[L12, L24]
Skin cancer	8	[L8, L12, L18, L34]
Bladder cancer	3	[L8]
Liver cancer	11	[L8, L9, L12, L13, L31, L34]
Colon/colorectal cancer	8	[L8, L9, L13, L16, L32]
Hypoxia	3	[L9, L13]
Head and neck cancer	2	[L9]
Oesophagus cancer/oesophageal squamous cell carcinoma	3	[L9]
Sarcoma cancer/Ewing sarcoma	2	[L9]
Types of cancer mentioned only once: stomach cancer [L8], α_v_β_3_ integrin (examination of tumour cells to survive during therapy), sentinel lymph node, neuroblastoma, cancer immunotherapy [L16], thymus cancer [L9], circulating cancer cells (CCC) [L9], cancer of unknown primary (CUP) [L9], oral cancer, small intestine cancer [L9], and pleural mesothelioma		

**Table 9:** Brain disorders.

	Number of Articles	Selected Articles
All articles concerning brain disorders/brain function	24	[L10, L11, L13, L14, L17]
Alzheimer’s disease	8	[L10]
Parkinson’s disease	9	[L13, L14]
Anxiety	2	[L11]
Regulates/chronic pain treatment	2	
Opioid receptor	3	
Substance use disorder	2	[L17]
Compulsive behaviour disorder like feeding disorders/obesity, gambling, addition	2	[L11]
Neurodegenerative	2	[L11]
Epilepsy	2	[L17]
Type of brain disorders/brain functions mentioned only once: neurological disorders, serotonin neurotransmitter, mood disorder, spinal cord injury, CNS-modulating agents, hallucinogens, Huntington’s disease, bipolar disorder, dementia, benzodiazepine receptors, monoamine oxidase-B (MAO-B), schizophrenia [L17], autism [L17], metabolic diseases [L11], and depression		

**Table 10:** Inflammation and infection.

	Number of Articles	Selected Articles
All articles concerning inflammation/infection	15	[L7, L13]
Osteomyelitis	3	[L7]
Neuroinflammation	4	
Bone infection	5	[L7, L13]
Rheumatoid arthritis	2	[L13]
Types of inflammation/infection mentioned only once: vascular inflammation, inflammatory joint disease, and arthritis.		
